# Oral Bisphosphonates for Colorectal Cancer Prevention: A Meta-Analytic Reappraisal Beyond Bone Health

**DOI:** 10.3390/jcm14113702

**Published:** 2025-05-25

**Authors:** Enrico Altiero Giusto, Rossella Donghia, Carlotta Giorgi, Paolo Pinton, Francesco Fiorica

**Affiliations:** 1 Azienda Ospedaliero-Universitaria ’S. Anna’, Via Aldo Moro 8, 44123 Cona, Italy; 2 Department of Medical Sciences, Section of Experimental Medicine, Laboratory for Technologies of Advanced Therapies, University of Ferrara, 44121 Ferrara, Italy; carlotta.giorgi@unife.it (C.G.); paolo.pinton@unife.it (P.P.); 3 Data Science Unit, National Institute of Gastroenterology—IRCCS “Saverio de Bellis”, Via Turi 27, 70013 Castellana Grotte, Italy; rossella.donghia@irccsdebellis.it; 4 Faculty of Medicine, Surgery and Prevention, University of Ferrara, Via Ludovico Ariosto 35, 44121 Ferrara, Italy; 5 Department of Clinical Oncology, Section of Radiation Oncology and Nuclear Medicine, AULSS 9 Scaligera, 37122 Verona, Italy; francesco.fiorica@aulss9.veneto.it; 6 Department of Clinical Oncology, Section of Medical Oncology, AULSS 9 Scaligera, 37122 Verona, Italy

**Keywords:** colon cancer, rectal cancer, colorectal cancer, bisphosphonate, drug therapy

## Abstract

**Background:** Oral bisphosphonates (BPs) are the standard therapy for osteoporosis and skeletal metastases, and exhibit anti-tumor properties in preclinical models. Observational studies assessing their impact on colorectal cancer (CRC) risk have yielded inconsistent results. We aimed to systematically review and meta-analyze the association between oral bisphosphonate use and CRC risk, applying a unified exposure definition. **Methods**: A systematic search was conducted in PubMed, Embase, and Scopus (January 1966–April 2025) to identify cohort, nested case–control, or population-based case–control studies reporting adjusted estimates of relative risk, odds ratios (ORs), or hazard ratios (HRs) for CRC among oral bisphosphonate users. Two reviewers independently screened studies, extracted data, and assessed quality using the Newcastle–Ottawa Scale. Random-effects meta-analyses pooled risk estimates for “any use” of bisphosphonates, with subgroup analyses by duration of use (<1, 1–3, >3 years). We assessed publication bias through Egger’s test and the trim-and-fill method. **Results:** A total of eight studies published between 2010 and 2020, including 29,169 CRC cases, fulfilled the inclusion criteria. Any bisphosphonate use was not significantly associated with CRC risk (pooled OR 0.97; 95% C.I., 0.90–1.03). However, 1–3 years of use conferred a protective effect (OR 0.86; 95% C.I., 0.73–0.99), as did >3 years (OR 0.91; 95% C.I., 0.85–0.97). Heterogeneity was moderate, and no significant publication bias was detected. **Conclusions**: While overall oral bisphosphonate exposure is not significantly linked to CRC risk, prolonged use (≥1 year) appears to reduce risk. Prospective studies and randomized trials are needed to confirm these chemo-preventive effects and guide clinical recommendations.

## 1. Introduction

Since receiving FDA approval in 1995, oral bisphosphonates (BPs) have been extensively used for a variety of medical indications over the past 16 years. These drugs are predominantly prescribed to treat and prevent osteoporosis in at-risk populations [1,2,3,4]. Additionally, bisphosphonates are used in the management of patients with bone metastases from various cancers, including primary gastrointestinal tumors, and are also being explored for the prevention of skeletal complications in cancer patients [1,2,5,6]. Recent studies have associated these drugs with a reduced risk of breast cancer [1,7]. BPs have the ability to activate both adaptive and innate immune responses and suppress tumor angiogenesis, invasion, and cell adhesion, thus influencing overall tumor progression [8]. Nitrogen-containing bisphosphonates interfere with the mevalonate pathway, leading to the inhibition of protein prenylation, which is believed to affect cancer cell growth and metastasis [8,9]. Furthermore, bisphosphonates have been shown to suppress colorectal cancer development in an experimental model of ulcerative colitis. Recent evidence also suggests that these drugs can reduce proliferation and induce apoptosis of colon cancer cells [8]. In the past 2 years, several studies have evaluated the association between oral bisphosphonate use and the risk of colorectal cancer, yielding conflicting results. The definition of bisphosphonate exposure varied among these studies, limiting the generalizability of their findings to routine clinical practice. This meta-analysis aims to further assess the existing evidence on the association between oral bisphosphonate use and colorectal cancer risk, and quantitatively synthesize the findings through a meta-analysis based on a standardized definition of bisphosphonate exposure.

## 2. Materials and Methods

We adhered to a standardized methodology [10] and present the findings in accordance with the Preferred Reporting Items for Systematic Reviews and Meta-Analyses (PRISMA) guidelines [11]. The meta-analysis is registered in PROSPERO n.1035664.

### 2.1. Search Strategy

To identify all pertinent papers, in English or other European languages, examining the risk of intestinal cancer associated with bisphosphonate (BP) therapy in well-defined observational studies, we performed a comprehensive and systematic search, using PubMed, Embase, and Scopus as search engines, from 1 January 1966 until 5 April 2025, using the MESH terms diphosphonates AND epidemiology, combined with a free-text search for the words “observational study” OR “case–control study” with the following filter: studies of humans (for the complete search strategy, see Supplementary Materials). In addition, reference lists of all included papers and their “related articles” were scrutinized to discover additional papers on the topic.

### 2.2. Inclusion/Exclusion Criteria

The full texts of potentially eligible articles were independently reviewed by two authors (E.A.G., and F.F.). We considered articles that were epidemiologic studies examining any intestinal cancer outcome, including colorectal, colon, or rectal cancer, in populations of any ethnicity, gender, or age in all countries and settings.

This meta-analysis includes studies that fulfilled the following criteria:

(1) Randomized controlled trials, prospective or retrospective cohort studies, nested case–control studies, or population-based case–control studies;
(2) The primary outcome was the incidence of colorectal, colon, or rectal cancer;
(3) The study reported relative risks (RRs), odds ratios (ORs), or hazard ratios (HRs), along with 95% confidence intervals or enough data to calculate them;
(4) Bisphosphonate exposure was defined as having at least one documented prescription or dispensation prescription of BPs, or reporting of BPs in a pharmacy record or an interview in at least the last two weeks.

Papers focused on cancer mortality and review articles were excluded. In instances of duplicate publications, the most recent study with the longest follow-up of patients was considered.

### 2.3. Data Extraction

Data extraction was performed independently by 2 authors (E.A.G. and F.F.). Disagreements were resolved by consensus. The included papers were reviewed in detail for data on the number of patients studied, the calendar year of publication, the number of colon and rectum cancers observed in the cohort, and the expected numbers in a matched background population, with relative confidence intervals at 95% (95% C.I.s).

We selected the ORs or HRs that provided the highest level of adjustment for potential confounders for inclusion in the primary analysis.

### 2.4. Quality Assessment

The quality of the studies was independently evaluated by two authors (E.A.G. and F.F.) using the 9-star Newcastle–Ottawa Scale (NOS) [12,13]. The NOS evaluates study quality based on three criteria: selection, comparability, and either exposure (for case–control studies) or outcome (for cohort studies). A maximum of four points is awarded for selection, two for comparability, and three for the assessment of exposure or outcome. A total score of nine points on the NOS indicates the highest methodological quality of the study.

### 2.5. Statistical Analysis

We presented the extracted random-effects estimates by means of forest plots displaying the association between BPs and CRC.

If ORs/HRs of CRC were reported without 95% C.Is, the interval was calculated assuming a gaussian distribution of cases.

The meta-analysis used the restricted maximum likelihood [14,15,16] (REML) for estimating variance components in mixed-effects models to estimate the between-study variance. The influence of the use of BP over time was assessed with the REML method.

Subsequently, pooled ORs with 95% CIs were calculated. Depending on the heterogeneity test, either a fixed- or a random-effects model was applied. To test the null hypothesis, the two-tailed probability level was set at 0.05. All analyses were conducted with Stata 18 (StataCorp. 2023. Stata Statistical Software: Release 18, College Station, TX, USA: StataCorp LLC.), and R: A Language and Environment for Statistical Computing (R Foundation for Statistical Computing, Vienna, Austria, 2024; https://www.R-project.org).

### 2.6. Ethics Statement

**Figure 1:**
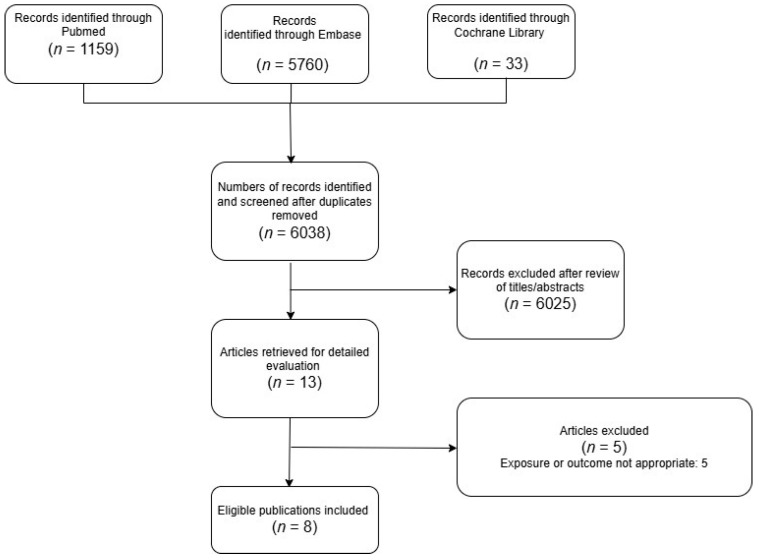
PRISMA selection progress.

This meta-analysis was conducted using data extracted from previously published population-based cohort studies and case–control studies (Figure 1). All included studies had obtained the necessary ethical approval from their respective institutional review boards, as documented in the original publications.

## 3. Results

### 3.1. Literature Search

The detailed steps of our literature search are shown in the Supplementary Materials. Briefly, we identified eight relevant articles [2,6,17,18,19,20,21,22] focusing on BP exposure and gastrointestinal cancers for inclusion in the meta-analysis.

### 3.2. Study Characteristics

Eight studies published between 2010 and 2020 examined the relationship between oral bisphosphonates and the risk of colorectal cancer (CRC). The detailed study characteristics are presented in Table 1.

There were three nested case–control studies: two were population-based case–control studies, and three were nationwide retrospective cohort studies. The studies were judged to warrant a score of 7 on the 9-star Newcastle–Ottawa Scale (see Supplementary Tables S1 and S2). The details of the quality assessment for each study are shown in the Supplementary Materials. Regarding exposure assessment, some studies, such as those by Green et al. [18], Ibanez et al. [19], Vinogradova et al. [21], and Vogtmann et al. [22], considered the number of written bisphosphonate prescriptions, while others, including those by Choi et al. [17], Rennert et al. [20], and Vestergaard [2], used pharmacy records of such prescriptions. Passarelli et al. employed an interview-based approach, where participants were asked to bring in all medications taken in the previous weeks.

**Figure 2:**
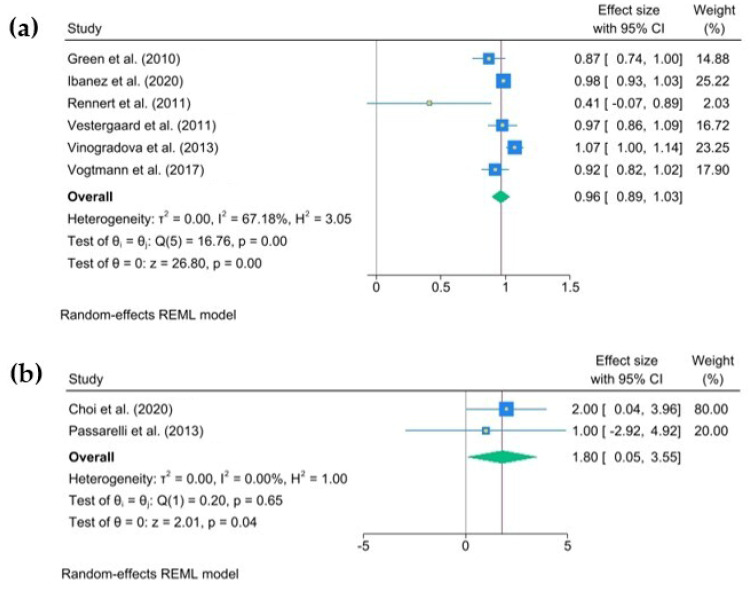
The pooled odds ratio for colorectal cancer associated with any use of oral bisphosphonates (BPs). The size of each square is proportional to the sample size of each study, with horizontal lines through the squares representing the 95% confidence intervals (C.I.s) for each study. In the pooled analysis, the diamond represents the pooled value, with the right and left ends of the diamond denoting the 95% C.I. for the analysis (**a**). The pooled hazard ratio for colorectal cancer associated with any use of oral bisphosphonates (BPs). The size of each square is proportional to the sample size for each study, and the horizontal lines through the squares represent the 95% C.I. for that study. For the pooled analysis, the diamond represents the pooled value, with the right and left ends of the diamond showing the 95% C.I. for the analysis (**b**) [2,6,17,18,19,20,21,22].

The multivariate-adjusted odds ratios (ORs) for colorectal cancer (CRC) risk for each individual study, as well as for all studies combined, comparing any bisphosphonate exposure with no exposure are presented in Figure 2a,b. The pooled joint OR was 0.96 (95% C.I., 0.89 to 1.03), heterogeneity: τ^2^ = 0.01, I^2^ = 67.18%, H^2^ = 3.05, test of qi = qj: Q(4) = 16.76, *p* = 0.00, test of q = 0: z = 26.8, *p* = 0.00. The pooled joint HR in the cohort studies was 1.80 (95% C.I., 0.05 to 3.55), heterogeneity: τ^2^ = 0.00, I^2^ = 0.00%, H^2^ = 1.00, test of qi = qj: Q(1) = 0.20, *p* = 0.65, test of q = 0: z = 2.01, *p* = 0.04. 

### 3.3. Duration of Exposure Analysis

**Figure 3:**
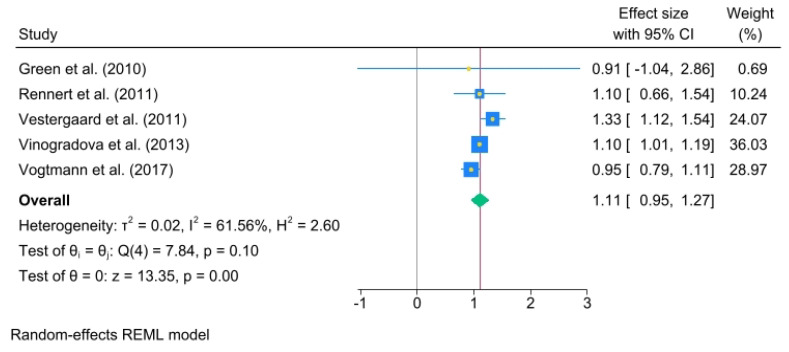
The pooled odds ratio for colorectal cancer associated with less than 1 year of oral bisphosphonate (BP) use. The size of each square is proportional to the sample size of each study, with horizontal lines through the squares representing the 95% confidence interval (C.I.) for each study. In the pooled analysis, the diamond represents the pooled value, with the right and left ends of the diamond indicating the 95% C.I. for the analysis [2,18,20,21,22].

The odds ratios (ORs) for each study and the pooled ORs for categories of use duration—less than 1 year, from 1 to 3 years, and more than 3 years—were analyzed. Less than 1 year of use of oral BPs yielded an OR = 1.11, (95% C.I., 0.95 to 1.27), heterogeneity: τ^2^ = 0.02, I^2^ = 61.56%, H^2^ = 2.60, test of qi = qj: Q(4) = 7.84, *p* = 0.10, test of q = 0: z = 13.35, *p* = 0.00 (Figure 3).

**Figure 4:**
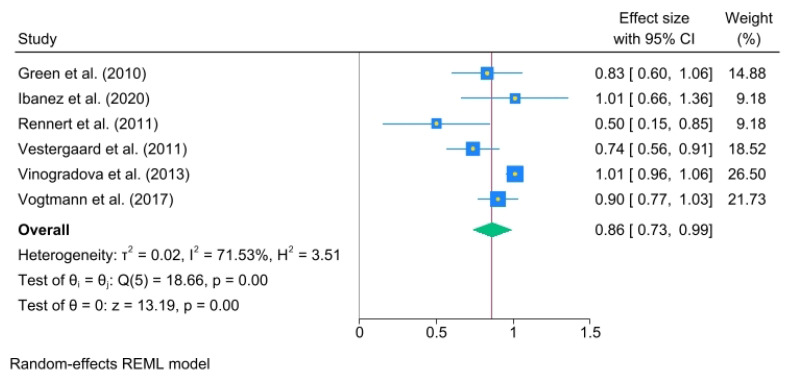
Oral bisphosphonate (BP) use for 1 to 3 years. The size of each square is proportional to the sample size of each study, with horizontal lines through the squares representing the 95% confidence interval (C.I.) for each study. In the pooled analysis, the diamond represents the pooled value, with the right and left ends of the diamond indicating the 95% C.I. for the analysis [2,18,19,20,21,22].

A significant association was noted between 1 and 3 years (OR, 0.86; 95% C.I., 0.73 to 0.99), heterogeneity: τ^2^ = 0.02, I^2^ = 71.53%, H^2^ = 3.51, test of qi = qj: Q(5) = 18.66, *p* = 0.00, test of q = 0: z = 13.19, *p* = 0.00 (Figure 4). 

**Figure 5:**
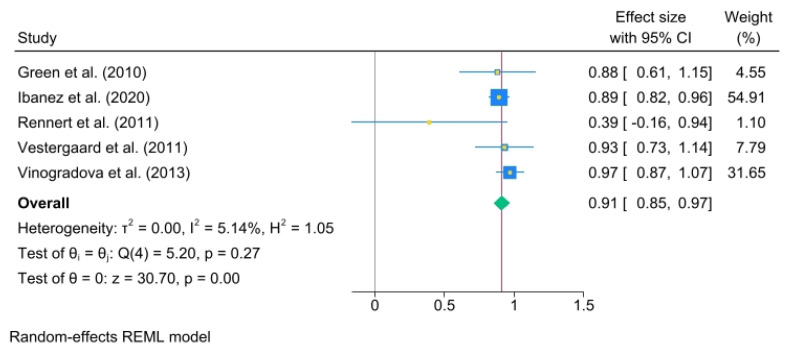
BP use for over 3 years. The area of each square is proportional to the sample size of the respective study, with horizontal lines through the squares representing the 95% confidence interval (C.I.) for that study. In the pooled analysis, the diamond symbol represents the pooled estimate, with the right and left points of the diamond indicating the 95% C.I. for the overall analysis [2,18,19,20,21].

The results for more than 3 years of use were as follows: (OR, 0.91; 95% C.I., 0.85 to 0.97) heterogeneity: τ^2^ = 0.00, I^2^ = 5.14%, H^2^ = 1.05, test of qi = qj: Q(4) = 5.20, *p* = 0.27, test of q = 0: z = 30.70, *p* = 0.00 (Figure 5).

### 3.4. Heterogeneity and Publication Bias

The Galbraith plot depicts the assessment of heterogeneity [23,24,25,26] and potential publication bias across the included studies. In order to estimate the origin of the heterogeneity, we considered whether quality was a problem in the included studies through a publication bias assessment.

**Figure 6:**
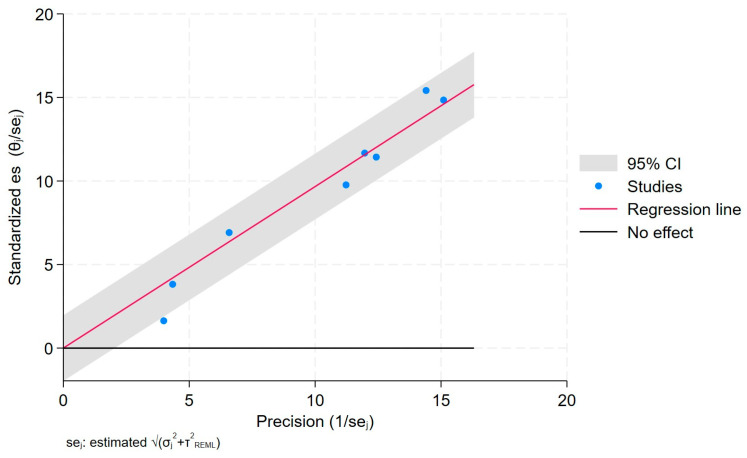
Galbraith plot.

This plot can help to visualize the extent of heterogeneity and identify any potential outlier studies that may have contributed to the observed heterogeneity (Figure 6).

Each study is represented as a point, where the x-axis denotes the precision (defined as the inverse of the standard error), and the y-axis indicates the standardized effect size. The red regression line represents the overall summary effect estimated from the random-effects model, and the gray shaded area corresponds to the confidence interval at 95% around regression line. The horizontal black line at y = 0 corresponds to the line of no effect, serving as a reference.

**Figure 7:**
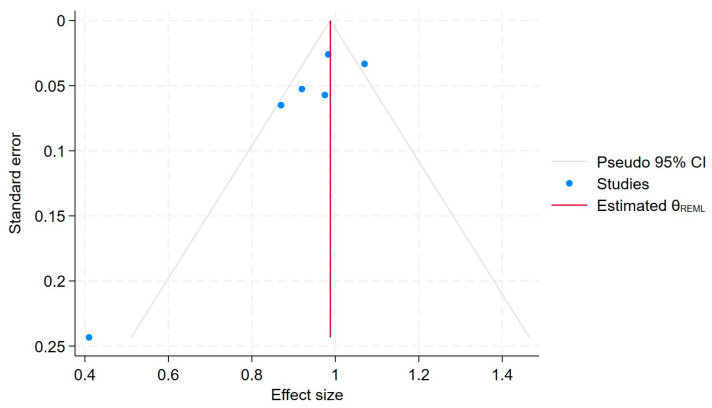
Funnel plot.

To further assess potential publication bias, we conducted the Egger regression test [11,27], which yielded a *p*-value of 0.08. This Egger test showed no statistically significant evidence of publication bias (*p* = 0.058). Additionally, the ORs and 95% C.I.s for any use of BPs and CRC did not change substantially with the “trim-and-fill method” of publication bias adjustment, suggesting that publication bias is not a major concern in this meta-analysis. A funnel plot for the publication bias [26,28] is also shown in Figure 7, which provides a visual representation of the potential asymmetry in the study effect sizes.

Each blue dot represents a single study, plotted with its effect size on the x-axis and its standard error on the y-axis. The vertical red line represents the overall effect estimate derived from the random-effects model using restricted maximum likelihood (REML). The pseudo 95% confidence interval region, shown as gray diagonal lines, outlines the expected spread of studies in the absence of bias or between-study heterogeneity.

## 4. Discussion

The current meta-analysis summarizes the results of eight large epidemiologic studies, including a total of 29,169 colorectal cancer cases. By incorporating data from a wide range of well-designed observational studies, this comprehensive analysis provides important insights into the potential association between bisphosphonate use and the risk of developing CRC.

This meta-analysis shows that overall, bisphosphonate exposure is not significantly associated with the risk of colorectal cancer. On the contrary, prolonged use for between 1 and 3 years and for over 3 years seems to have a protective effect against colorectal cancer.

In their primary studies, Choi et al. [17], Ibanez-Sanz et al. [19], Passarelli et al. [6], and Vogtmann et al. [22] all reported no statistically significant association between bisphosphonate use and CRC. Similarly, Green et al. [18] and Vestergaard [2] found a borderline, insignificant risk reduction for CRC with the use of oral treatment, whereas Rennert reported a significant risk reduction for CRC.

Interestingly, the nationwide retrospective cohort studies by Choi et al. [17], Passarelli et al. [6], and Vestergaard [2] showed an overall not statistically significant association with CRC.

Bisphosphonates are synthetic compounds categorized into older non-nitrogen-containing and newer nitrogen-containing types. These drugs have exhibited concentration-dependent direct anti-cancer effects against various malignancies, including colorectal cancer, through mechanisms including the inhibition of angiogenesis, prevention of tumor progression, induction of tumor cell apoptosis, and suppression of metastasis [5,18]. Bisphosphonates have been shown to inhibit the growth, proliferation, and metastasis of colorectal cancer cells, and may also work synergistically with chemotherapeutic agents to enhance their anti-cancer effects [18,22,29]. The anti-cancer activities of bisphosphonates are believed to stem from their ability to disrupt the mevalonate pathway, which is crucial for cancer cell growth and metastasis, as well as their capacity to inhibit angiogenesis and tumor cell adhesion and promote apoptosis [8]. However, the precise mechanisms by which bisphosphonates may reduce the risk of colorectal cancer require further investigation, and additional research is needed to confirm these findings and determine the optimal use of bisphosphonates for cancer prevention. Although experimental studies have demonstrated the ability of amino bisphosphonates to promote apoptosis and suppress growth and angiogenic factors, it remains unclear whether they play a role in the primary prevention of cancer [5,9].

Bisphosphonate treatment has also been associated with adverse outcomes such as osteonecrosis of the jaw and atypical femoral fractures. Osteonecrosis of the jaw has mainly been observed in patients with particular risk factors, including the use of high-dose intravenous bisphosphonate therapy, exposure lasting more than 1 year, concurrent cancer and anti-cancer treatments, pre-existing dental conditions, dental implants, and smoking [1,2,3,4,17]. Although the absolute risk is low, prolonged bisphosphonate use for over 5 years can lead to a modest risk of atypical femoral fractures in younger women. Further prospective studies are required to identify the subgroups at highest risk. Additional research is also needed to evaluate the long-term adverse effects and risks associated with extended bisphosphonate therapy, and to determine whether the therapeutic benefits truly outweigh the associated risks. While the overall risk of these adverse events is relatively low, it is important for healthcare providers to carefully evaluate the potential risks and benefits of bisphosphonate therapy for each individual patient, especially those with additional risk factors. Ongoing monitoring and appropriate management strategies are crucial to minimize the likelihood of these complications and optimize the safety and efficacy of bisphosphonate treatment.

Given the increasing global burden of colorectal cancer and the well-established safety profile of bisphosphonates for their approved indications, further high-quality research, including randomized controlled trials, is imperative to definitively evaluate their potential as a chemo-preventive strategy.

Meta-analysis is a crucial tool that helps to clarify the reasons behind discrepancies in trial results, enhances research and editorial standards by highlighting the strengths and weaknesses of the existing body of evidence, and provides practitioners with an objective perspective on the research literature [23,30]. The present meta-analysis offers several advantages. First, we employed a consistent definition of exposure, reanalyzing the data based on this standardized definition before pooling it for the analysis. Second, the large number of total cases and controls enhanced the statistical power of the analysis. Thirdly, we used restricted maximum likelihood methods to analyze our data, obtaining the maximal likelihood estimate of variance components to maximize the precision of the data. Fourthly, we performed subgroup analysis based on the duration of bisphosphonate exposure to explore potential effect modifications. Fifthly, we used data with the greatest adjustment.

This meta-analysis has different limitations that warrant consideration. Firstly, as an observational study design, it is inherently limited in its ability to fully account for potential confounding factors present in the included studies. Although the primary studies attempted to adjust for relevant confounding variables, the possibility of residual or unknown confounding factors influencing the observed findings cannot be entirely eliminated, necessitating some caution in the interpretation of the results. Furthermore, while major publication bias was not detected in this analysis, the potential for such bias cannot be entirely ruled out. Small studies with null results are generally less likely to be published, while small studies with larger effects have a higher chance of being published, potentially introducing publication bias. Furthermore, the problem of exposure misclassification could be create bias in the analysis. The Egger regression test is generally considered more robust than the rank correlation test for evaluating small study effects, but both have limited power unless significant bias is present, and the number of studies included is sufficiently large. Therefore, the results of this meta-analysis should be interpreted while bearing these important limitations in mind.

## 5. Conclusions

The findings from this meta-analysis suggest that prolonged use of oral bisphosphonates may be associated with a reduced risk of colorectal cancer. However, this potential protective effect was not observed for less than 1 year of bisphosphonate use. Further in-depth longitudinal studies are necessary that consider all potential confounding factors, examine different types and methods of bisphosphonate use, and evaluate the effects of cumulative dose and duration of use on the risk of both colorectal and esophageal cancer. This approach would provide more accurate estimates and a clearer understanding of the potential role of bisphosphonates in gastrointestinal carcinogenesis. Ultimately, the results of these observational studies will need to be validated through large, well-conducted randomized clinical trials or secondary analysis of previous randomized trials on oral bisphosphonates and osteoporosis, with long-term clinical follow-up to definitively assess the relationship between bisphosphonate use and gastrointestinal cancer risk.

## Figures and Tables

**Table 1:** Characteristics of included studies and availability of data on patients with CRC receiving oral BPs.

Author, Year	Study Design	Mean Age (yrs)	No. Control	No. Cases	Estimation and 95% C.I.	Study Quality	Adjustment for Covariates	Exposition Assessment
Choi et al. (2020) [17]	Nationwide retrospective cohort study	Control: 67.10 Cases: 68.90	402	59	HR: 1.05 (0.80–1.38) OR: 1.05 (0.80–1.38)	7	Age, smoking, alcohol consumption, household income, number of received upper GI endoscopies (only of stomach cancer), number of received lower GI endoscopies (only of colorectal cancer), systolic blood pressure, total cholesterol, and fasting serum glucose	Pharmacy record of filling of a prescription
Green et al. (2010) [18]	Nested case–control study	Control: 70.10 Cases: 70.10	51,467	10,365	OR: 0.87 (0.77–1.00)	7	Age, BMI, smoking status (never, past, current, missing), alcohol intake, prescription of NSAIDs as aspirin, prescription of corticosteroids, prescription of acid-suppressant drugs	One or more prescriptions of oral BPs
Ibanez et al. (2020) [19]	Nested case–control study	Control: 75.70 Cases: 75.50	15,289	2941	OR: 0.98 (0.93–1.03)	7	BMI, ethnicity, family history of CRC, self-reported sports activity, vegetable intake (≥5 portions per day), aspirin use once a day for at least 3 years, statin use for ≥1 year, use of postmenopausal hormones, calcium	One or more dispensations of oral BPs on prescription records
Passarelli et al. (2013) [6]	Nationwide retrospective cohort study	--	1805	126	HR: 0.88 (0.72–1.72) OR: 0.88 (0.72–1.72)	7	Age, gender, region, year, socioeconomic status, body mass index, smoking, alcohol, comorbidities, nonsteroidal anti-inflammatory drugs, propensity score, bisphosphonates, and vitamin D	Interview to which participants were instructed to bring medication bottles/packaging for drugs taken at least twice per week during previous 2 weeks
Rennert et al. (2011) [20]	Nationwide retrospective cohort study	Control: 72.00 Cases: 71.10	933	933	OR: 0.41 (0.25–0.67)	7	BMI, ethnicity, family history of CRC, self-reported sports activity, vegetable consumption (≥5 portions per day), aspirin use once a day for at least 3 years, statin use ≥ 1 year, use of postmenopausal hormones, calcium	Pharmacy record of filling of a prescription
Vestergaard et al. (2011) [2]	Nationwide retrospective cohort study	Control: 70.50 Cases: 70.50	--	409	OR: 0.97 (0.87–1.09)	7	Age, gender, use of inhaled corticosteroids and β-agonist as proxy for smoking, alcoholism, antacid drugs, aspirin, and NSAIDs	Pharmacy record of filling of a prescription
Vinogradova et al. (2013) [21]	Nested case–control study	Control: 71.60 Cases: 71.50	8576	1831	OR: 1.07 (1.00–1.14)	7	BMI, smoking status, alcohol consumption, ethnicity, rheumatoid arthritis, osteoporosis and fractures, use of other osteoporosis drugs, vitamin D, NSAIDs, corticosteroids, acid-lowering drugs, years of data, and family history of colorectal cancer, diabetes, colitis, and Crohn’s disease	One or more dispensations of oral BPs on prescription records
Vogtmann et al. (2017) [22]	Nested case–control study	Control: 65.94 Cases: 66.40	599,534	12,505	OR: 0.92 (0.83–1.02)	7	Gender, age at time of index date (+/−2 years), duration of membership prior to index date (+/−1 year), race, and region of residence. Adjusted model additionally included age, smoking, alcohol use, Charlson comorbidity index, use of NSAIDs, and previous lower GI endoscopy	One or more prescriptions of oral BPs

Abbreviations: OR, odds ratio; HR, hazard ratio; 95% C.I., confidence interval at 95%; BPs, bisphosphonates; GI, gastrointestinal; BMI, body mass index; NSAIDs, nonsteroidal anti-inflammatory drugs; CRC, colorectal cancer.

## Data Availability

The data, analytic methods, and study materials used in this research will be made available to other researchers upon request.

## Supplementary Materials

The following supporting information can be downloaded at https://www.mdpi.com/article/10.3390/jcm14113702/s1: Table S1: Quality assessment for studies using the Newcastle–Ottawa Quality Assessment Scale: case–control studies; Table S2: Quality assessment for studies using the Newcastle–Ottawa Quality Assessment Scale: cohort studies.

## Abbreviations

The following abbreviations are used in this manuscript:

BP	Bisphosphonate
CRC	Colorectal cancer
FDA	Food and Drug Administration
