# Supplementary material for: Oral Bisphosphonates for Colorectal Cancer Prevention: A Meta-Analytic Reappraisal Beyond Bone Health
Source: J Clin Med. 2025 May 25;14(11):3702. doi: 10.3390/jcm14113702 (PMC12155516; doi:10.3390/jcm14113702)
Supplement: Supplementary file 1 [file jcm-14-03702-s001.zip › jcm-3642986-supplementary.pdf]

Supplementary

Search Strategy

PUBMED

("Diphosphonates"[Mesh] OR diphosphonates[All Fields] OR diphosphonate[All Fields] OR "Bisphosphonates"[All Fields] OR "Alendronate"[Mesh] OR alendronate[All Fields] OR "Clodronic acid"[Mesh] OR clodronic acid[All Fields] OR clodronate[All Fields] OR "Etidronic acid"[Mesh] OR etidronic acid[All Fields] OR etidronate[All Fields] OR "Ibandronic acid"[Substance Name] OR ibandronic acid[All Fields] OR ibandronate[All Fields] OR Minodronate[All Fields] OR "YM 529"[Substance Name] OR "YM 529"[All Fields] OR Neridronate[All Fields] OR "6-amino-1-hydroxyhexane-1,1-diphosphonate"[Substance Name] OR "6-amino-1-hydroxyhexane-1,1-diphosphonate"[All Fields] OR Olpadronic acid[Substance Name] OR olpadronic acid[All Fields] OR olpadronate[All Fields] OR Pamidronate[Substance Name] OR pamidronate[All Fields] OR Risedronic acid[Substance Name] OR risedronic acid[All Fields] OR risedronate[All Fields] OR Tiludronic acid[Substance Name] OR tiludronic acid[All Fields] OR tiludronate[All Fields] OR Zoledronic acid[Substance Name] OR zoledronic acid[All Fields] OR zoledronate[All Fields]) AND ("Observational studies"[All Fields] OR "Cohort studies"[All Fields] OR "Case control studies"[All Fields] OR "case-control studies"[All Fields] OR "Case referent studies"[All Fields] OR "case-referent studies"[All Fields]) AND Humans[Mesh]

EMBASE

('bisphosphonate'/exp OR 'bisphosphonate' OR 'diphosphonate'/exp OR 'diphosphonate' OR 'alendronate'/exp OR 'alendronate' OR 'clodronate'/exp OR 'clodronate' OR 'etidronate'/exp OR 'etidronate' OR 'ibandronate'/exp OR 'ibandronate' OR 'minodronate'/exp OR 'minodronate' OR 'neridronate'/exp OR 'neridronate' OR 'olpadronate'/exp OR 'olpadronate' OR 'pamidronate'/exp OR 'pamidronate' OR 'risedronate'/exp OR 'risedronate' OR 'tiludronate'/exp OR 'tiludronate' OR 'zoledronate'/exp OR 'zoledronate') AND ('observational study'/exp OR 'observational study' OR 'cohort analysis'/exp OR 'cohort analysis' OR 'case control study'/exp OR 'case control study' OR 'case referent study') AND [humans]/lim

COCHRANE LIBRARY

diphosphonate OR diphosphonates OR bisphosphonates OR alendronate OR clodronic acid OR clodronate OR etidronic acid OR etidronate OR ibandronic acid OR ibandronate OR minodronate OR neridronate OR "6-amino-1-hydroxyhexane-1,1-diphosphonate" OR olpadronic acid OR olpadronate OR pamidronate OR risedronic acid OR risedronate OR tiludronic acid OR tiludronate OR zoledronic acid OR zoledronate

Quality Assessment for Studies

	Green et al.	Rennert et al.	Ibanez et al.	Vinogradova et al.	Vogtmann et al.
**Selection**					
1. Is the case definition adequate?					
a. Yes, with independent validation ★	*	*	*	*	*
b. Yes (e.g., record linkage or based on self-reports)					
c. No description					
2. Representativeness of the cases					
a. Consecutive or obviously representative series of cases★	*	*	*	*	*
b. Potential for selection biases or not stated					
3. Selection of controls					
a. Community controls ★	*	*	*	*	*
b. Hospital controls					
c. No description					
4. Definition of controls					
a. No history of disease (end point) ★	*	*	*	*	*
b. No description of source					
**Comparability**					
1. Comparability of cases and controls on the basis of the design or analysis					
a. Study controls for CRC cases ★					
b. Study controls for any additional factor	*	*	*	*	*
**Exposure**					
1. Ascertainment of exposure					
a. Secure record (e.g., surgical records) ★	*	*	*	*	*
b. Structured interview where blind to case/control status ★					
c. Interview not blinded to case/control status					
d. Written self-report or medical record only					
e. No description					
2. Same method of ascertainment for cases and controls					
a. Yes ★	*	*	*	*	*
b. No					
3. Non-response rate					
a. Same rate for both groups ★					
b. Non-respondents described					
c. Rate different and no designation					

Supplementary Table S1. Quality assessment for studies using the Newcastle–Ottawa Quality Assessment Scale: case-control studies.

	Choi et al.	Passarelli et al.	Vestergaard et al.
**Selection**			
1. Representativeness of the exposed cohort			
a. Truly representative of the average population ★	*	*	*
b. Somewhat representative of the population ★			
c. Selected group of users (e.g., nurses, volunteers)			
d. No description of the derivation of the cohort			
2. Selection of the nonexposed cohort			
a. Drawn from the same community as the exposed cohort ★	*	*	*
b. Drawn from a different source			
c. No description of the derivation of the nonexposed cohort			
3. Ascertainment of exposure			
a. Secure record (e.g., surgical records) ★	*		*
b. Structured interview ★		*	
c. Written self-report			
d. No description			
4. Demonstration that outcome of interest was not present at start of study			
a. Yes ★	*	*	*
b. No			
**Comparability**			
1. Comparability of cohorts on the basis of the design or analysis			
a. Study controls for colorectal cancer ★			
b. Study controls for any additional factor★	*	*	*
**Outcome**			
1. Assessment of outcome			
a. Independent blind assessment ★			
b. Record linkage ★	*	*	*
c. Self-report			

d. No description			
2. Was follow-up long enough for outcomes to occur?			
a. Yes ★	*	*	*
b. No			
3. Adequacy of follow-up of cohorts			
a. Complete follow-up: all subjects accounted for ★	*	*	*
b. Subjects lost to follow-up unlikely to introduce bias/small number lost/> 10 % follow-up, or description provided of those lost) ★			
c. Follow-up rate ≤ 10% and no description of those lost			
d. No statement			

Supplementary Table S2. Quality assessment for studies using the Newcastle–Ottawa Quality Assessment Scale: cohort studies.

NOTE: For each study, a maximum of one star for each numbered item within the Selection and Exposure categories was awarded. A maximum of two stars was given for Comparability. The total number of stars earned (maximum score of 9) was a measure of study quality.
